# Association of Prostate-Specific Antigen With Age, Digital Rectal Examination, and Lower Urinary Tract Symptoms in the Lebanese Population: A Cross-Sectional Study

**DOI:** 10.7759/cureus.66991

**Published:** 2024-08-16

**Authors:** Yehya Tlaiss, Marc Jreij, Mohamad Tlais, Zahi F Yammine, Aziz M Najjar, Rania Naoufal, Hanadi Samaha, Marwan Najjar, Imad Ghantous

**Affiliations:** 1 Biostatistics and Epidemiology, Saint George Hospital University Medical Center, Beirut, LBN; 2 Urology, Saint George Hospital University Medical Center, Beirut, LBN; 3 Laboratory Medicine, Saint George Hospital University Medical Center, Beirut, LBN; 4 Rheumatology, Saint George Hospital University Medical Center, Beirut, LBN

**Keywords:** lebanese population, prostate cancer (pca), digital rectal examination (dre), lower urinary tract symptoms (luts), prostate-specific antigen (psa)

## Abstract

Background: Prostate cancer (PCa) is a leading cause of mortality in men worldwide. Prostate-specific antigen (PSA) testing is a standard method for PCa detection, yet its association with age, digital rectal examination (DRE) results, and lower urinary tract symptoms (LUTS) remains understudied, particularly in the Lebanese population.

Objective: This study aimed to investigate the association of PSA levels with age, DRE results, and LUTS severity among Lebanese men.

Methods: A total of 725 men aged 55-70 years were recruited from a men's health campaign at Saint George Hospital University Medical Center in Lebanon. PSA levels, DRE results, and International Prostate Symptom Score (IPSS) were assessed. Statistical analysis included Kruskal-Wallis tests and Spearman's rho correlation coefficient.

Results: Participants exhibited a significant correlation between age and PSA levels (r = 0.138, p < 0.01). PSA levels varied significantly across age groups (p = 0.029), with higher mean PSA levels observed in older age groups. IPSS status correlated positively with PSA levels (r = 0.23, p < 0.001), indicating higher PSA levels associated with increased LUTS severity. Abnormal DRE findings were significantly associated with elevated PSA levels (p < 0.00), suggesting their potential as an indicator of prostate abnormalities.

Conclusion: This study highlights the importance of age-specific reference ranges for PSA levels in the Lebanese population. Elevated PSA levels were associated with older age, increased LUTS severity, and abnormal DRE findings. These findings highlight the significance of integrating PSA testing with clinical assessments for PCa detection and risk stratification in Lebanon.

## Introduction

Prostate cancer (PCa) is the most common cause of death in men, second only to lung cancer, and one of the most common types of cancer in men overall [1]. Epithelial cells of the prostate gland produce prostate-specific antigen (PSA) after its androgenic receptors are activated. Though limited in specificity and sensitivity, measuring the PSA level has been a staple of early detection and screening for PCa over the past three decades. This is mainly due to the increase in PSA levels with prostatic hyperplasia [1]. Notably, several factors such as smoking, race, geographical location, weight, and age have been found to influence PSA levels [1]. The correlation of serum PSA level to variables such as age and lower urinary tract symptoms (LUTS), as well as its use in conjunction with digital rectal examinations (DRE) for diagnosis, has been well established [1,2]; however, all these relationships have been notoriously under-investigated in the Middle East and Lebanon in particular. While PSA level interpretation may vary by age, symptoms, and DRE, the specific context of these factors has not yet been studied within the Lebanese population. To that end, we recruited 725 patients in a men’s health campaign at Saint Georges University Hospital in Beirut, Lebanon to gather data on serum PSA levels, DRE results, and LUTS, and investigated any correlations that may exist between these variables with the age groups of our patients. This study not only fills a significant gap in regional data but also contributes to the global understanding of PSA testing and its implications, offering insights that could enhance the effectiveness of PCa screening and diagnosis strategies in diverse populations worldwide. In Lebanon, the population exhibits unique characteristics due to genetic, environmental, and lifestyle factors that may differ from those observed in Western populations. Additionally, Lebanon's healthcare system presents significant disparities in access to care, particularly in rural areas, where preventive services such as PSA testing may not be readily available. Moreover, varying levels of health literacy in the country further complicate the timely and effective detection of PCa. This study aims to fill the gap in understanding how these factors specifically affect PSA levels and prostate health in the Lebanese male population.

PSA and age

The relationship between age and PSA levels is well-established in the literature worldwide. Oesterling et al. [3] famously developed the first age-specific reference range for PSA level and found a direct positive correlation between PSA level and age. Prior to these findings, the normal upper limit for PSA level 4.0 ng/mL was used as a single cut-off for all ages [4]. Similarly, studies conducted in Iran reinvestigating that relation found the same positive correlation between PSA levels and age [1]. Specifically, in Reza et al.’s [1] study, the PSA levels (95th percentile) were found to be “0.9 (0-4.89) ng/mL in the age group of 60-64 years, 1.1 (0-4.88) ng/mL in the age group of 65-69 years, 0.93 (0-9.01) ng/mL in the age group of 70-74 years, 1.3 (0-7.95) ng/mL in the age group of 75-79 years, 1.9 (0-11.98 ng/mL) in the age group of 80-84 years, and 1.45 (0-33.17) ng/mL in the 85 and older group.” Moreover, the lack of an age-specific reference range for PSA levels in Lebanese men makes investigating that variable more compelling.

PSA and DRE

The reliance on DRE as the sole diagnostic tool for PCa diagnosis has dwindled over the years. Prior to the introduction of PSA testing in the mid-1980s, DRE was the primary method of diagnosing PCa [5]. Current guidelines concerning the use of DRE for PCa diagnosis are conflicting. While the National Institute for Health and Care Excellence (NICE) suggests that DRE is sufficient as a referral standard for PCa suspicion, guidelines from the National Comprehensive Cancer Network (NCCN) propose DRE solely for men with high PSA [6,7]. Conversely, the American Urological Association (AUA) denies the presence of evidence for DRE utility altogether [8]. Sajjad et al. [9] argue that the fixation on DRE use in these guidelines can be attributed to the outdated nature of the data and the lack of newly founded evidence supporting DRE use in the post-MRI era of PCa diagnosis. Their assessment of the value of DRE as a referral criterion concluded that PSA testing should be used to redirect men with an abnormal DRE to MRI directly instead of repeating DRE. To support the concurrent use of PSA testing with DRE, a retrospective study of 35,350 patients who underwent DRE screening showed prognostic efficacy when PSA level was higher than 3 ng/ml and marginal to limited usefulness at less than 3 ng/ml [10]. Similarly, a rare study on an Arab population found that a positive DRE with high PSA makes PCa more likely [11]. Few studies have explored the direct statistical relationship between positive DRE and PSA levels [5,11]. To that end, our study aims to investigate a direct statistical correlation between positive DREs and PSA levels in different age groups.

PSA and LUTS

Studies have shown that PCa is asymptomatic during the early stages [11,12]. Symptoms may only appear after tumor compression of the urethra, sphincter, or neurovascular bundle [12]. Symptoms of PCa range from hematuria and erectile dysfunction to LUTS such as urinary frequency, hesitancy, nocturia, and terminal dribble. Notably, LUTS are very common among men older than 60 years old and are found at about 80% in that age group [13]. Moreover, conditions other than cancer like prostatitis and benign prostatic hyperplasia (BPH) can present with LUTS [14]. Furthermore, it was found that the severity of LUTS cannot predict the exact staging of PCa nor the presence of the cancer altogether [15]. This indicates that LUTS are not specific to PCa given how common they are in older men and other indolent cases. While the NICE suggests conducting PSA testing and DREs in men with LUTS [1,16], concerns were raised regarding overdiagnosis and/or overtreatment for benign conditions [17-19]. Nonetheless, screening with PSA and DRE is still considered the gold standard for first-line diagnosis before referral to more specific/sensitive tests like biopsies and multiparametric MRI [20,21]. Though it was established that men with LUTS may have elevated PSA levels, that could be due to various non-cancerous reasons as discussed above. Therefore, the data on direct correlations between PSA levels and LUTS severity are limited due to the foggy relationship between the two. The study done by Turk & Un [22] showed that as LUTS severity increases from mild to severe, PSA levels and prostate volume increase. However, the study suffers from a small sample size and non-specific age groups. Similarly, a study conducted on Ghanaian men found a statistically significant positive association (p < 0.05) between LUTS and PSA levels, prostate volume, and age [23]. Given the limited data on direct correlations between LUTS and PSA, especially in the context of specific age groups, our study included a thorough exploration of the two variables in addition to definitive age group structure as per the European Association of Urology (EAU) recommendations [24].

Objective

This study aims to examine the association of PSA levels with factors such as age, LUTS severity, and DRE results, all within the Lebanese population. The study hypothesizes a positive correlation between PSA levels with age, a positive correlation between PSA levels and LUTS severity, and a positive correlation between PSA levels and positive DRE results.

## Materials and methods

Study setting and participants

The study was conducted at the Saint George Hospital University Medical Center (SGHUMC) in Lebanon during the month of November 2022. The campaign targeted men aged between 55 and 70 years who lacked medical coverage in Lebanon. Inclusion criteria included all consenting men attending the campaign who were within the age range, while men with prior PCa or surgery were excluded. Participants were recruited from the Primary Health Care Center at SGHUMC.

Data collection

The design of this study was a cross-sectional analysis. Participants underwent a comprehensive physical examination, during which their International Prostate Symptom Score (IPSS) status was assessed using a standardized questionnaire. DRE was conducted by trained urologists. Blood tests were performed to measure PSA levels. All data were recorded in the hospital's patient management system, utilizing the Oracle platform.

Potential confounding variables included smoking and family history of PCa. Patients were asked about their smoking habits and family history of PCa right after the IPSS questionnaire.

Biases

There are two potential biases of note that were addressed. Firstly, selection bias, which was resolved by the random sampling of the campaign patients. Secondly, measurement bias, which was minimized through the use of standardized data collection protocols like the IPSS for LUTS severity. The only concern remains the reliability of DRE tests as they were conducted by different urologists. Though the urologists in our study were thoroughly trained in DRE exams, the results might differ between each urologist’s subjective assessment.

Statistical analysis

Quantitative variables, including age and PSA levels, were described using summary statistics such as the mean and standard deviation. Age was categorized into several groups to facilitate analysis, and the main research variables were described based on this categorization.

The collected data were analyzed using SPSS version 26 (IBM Corp., Armonk, NY). Statistical tests employed included the Kruskal-Wallis test for comparisons between groups as well as Spearman's rho correlation coefficient to assess associations between variables. Results were presented in tables and diagrams.

A p-value of less than or equal to 0.05 was considered statistically significant. For multiple comparisons, a more stringent significance level (e.g., p < 0.01) could be considered to account for potential type I errors.

## Results

A total of 725 participants were included in the study. The age of participants ranged from 47 to 81 years, with a mean age of 62.5 years and a standard deviation of 4.9 years. The distribution of participants across age groups revealed that a small proportion of individuals (0.1%, N = 1) were aged less than 50 years, while 16 (2.1%) fell within the age group of 50 to 54 years. The majority of participants were distributed across the age groups of 55 to 59 years (27.2%, N = 209), 60 to 64 years (30.9%, N = 237), and 65 to 69 years (25.9%, N = 199). A smaller proportion of participants (8.7%, N = 63) were aged above 70 years (Table 1).

**Table 1:** Characteristics of SGHUMC men's health campaign beneficiaries. Note: The data are represented as N (%). SGHUMC: Saint George Hospital University Medical Center; IPSS: International Prostate Symptom Score.

Investigated variables	Number (N)	Percent (%)
Age (years)		
50 - 54	16	2.1%
55 - 59	209	27.2%
60 - 64	237	30.9%
65 - 69	199	25.9%
Above or equal to 70	63	8.7%
IPSS status		
Mildly symptomatic	346	47.6%
Moderately symptomatic	276	38.0%
Severely symptomatic	103	14.3%
Total	725	100.0%

**Figure 1:**
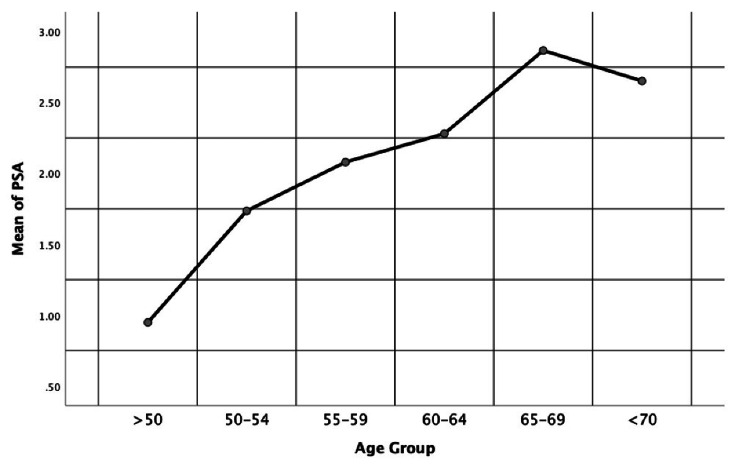
Mean serum PSA levels (ng/mL) according to age groups. Note: The data are represented as mean ± SD. PSA: prostate-specific antigen.

The Kruskal-Wallis test of PSA levels across age groups (Table 2) revealed a significant variation (p = 0.029). Participants aged 65-69 years exhibited the highest mean PSA level of 2.87 ng/mL, followed closely by those above 70 years old, with a mean PSA level of 2.65 ng/mL. Conversely, participants in the other age groups showed progressively lower mean PSA levels, ranging from 1.7358 ng/mL to 2.2805 ng/mL. These findings suggest a potential association between age and PSA levels. Generally, the mean serum PSA level of the beneficiaries had increased in different age groups with the increase in age (Figure 1).

**Table 2:** Distribution of serum PSA levels according to age group. Note: The data are represented as mean ± SD. A p-value of less than or equal to 0.05 was considered statistically significant. PSA: prostate-specific antigen.

Age group	N	PSA mean (mean ± SD)	Std. deviation	Std. error	95% CI lower bound	95% CI upper bound	Min	Max	p-value at 95% CI
50 - 54	16	1.74 ± 1.52	1.52	0.38	0.92	2.55	0.54	6.39	
55 - 59	209	2.08 ± 2.10	2.10	0.15	1.79	2.37	0.01	15.81	
60 - 64	237	2.28 ± 2.65	2.65	0.17	1.94	2.62	0.01	23.98	
65 - 69	199	2.87 ± 3.38	3.38	0.24	2.39	3.34	0.14	31.15	
Above 70	63	2.65 ± 2.46	2.46	0.31	2.03	3.27	0.25	15.50	
Total	724	2.40 ± 2.71	2.71	0.10	2.20	2.60	0.01	31.15	0.029

The Spearman's rho correlation analysis revealed a statistically significant positive correlation between age and PSA levels (r = 0.138, p < 0.01). This finding indicates that as age increases, there tends to be an increase in PSA levels among the participants.

The Kruskal-Wallis test conducted to examine the relationship between the IPSS status groups and PSA levels yielded significant findings (p = 0.00) (Table 3). Mean comparisons revealed that PSA levels varied significantly across different IPSS status groups. Specifically, participants classified as severely symptomatic had a significantly higher mean PSA level (M = 4.06 ng/mL, SD = 3.63) compared to those categorized as mildly symptomatic (M = 2.17 ng/mL, SD = 2.62) and moderately symptomatic (M = 2.07 ng/mL, SD = 2.16).

**Table 3:** Distribution of serum PSA levels according to IPSS status. Note: The data are represented as mean ± SD. A p-value of less than or equal to 0.001 was considered statistically significant. IPSS: International Prostate Symptom Score; PSA: prostate-specific antigen.

IPSS status	N	PSA mean (mean ± SD)	Std. deviation	Std. error	95% CI lower bound	95% CI upper bound	Min	Max	p-value at 95% CI
Mildly symptomatic	346	2.17 ± 2.62	2.62	0.14	1.89	2.44	0.01	31.15	
Moderately symptomatic	275	2.07 ± 2.16	2.16	0.13	1.81	2.33	0.01	15.50	
Severely symptomatic	103	4.06 ± 3.63	3.63	0.36	3.36	4.77	0.20	23.98	
Total	724	2.40 ± 2.71	2.71	0.10	2.20	2.60	0.01	31.15	0.00

**Figure 2:**
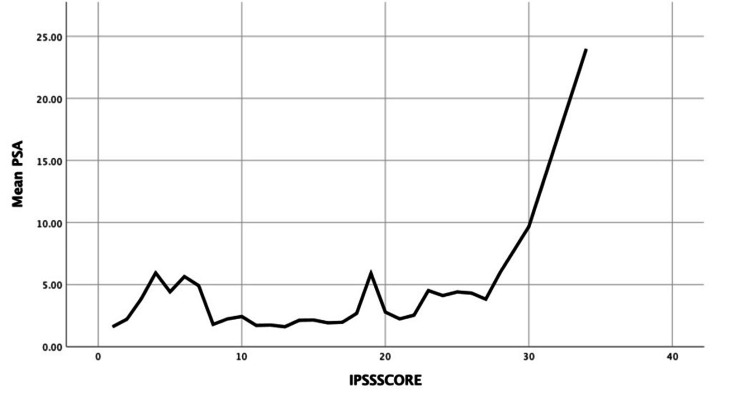
Mean serum PSA levels (ng/mL) according to the IPSS score. Note: The data are represented as mean ± SD. IPSS: International Prostate Symptom Score; PSA: prostate-specific antigen.

The Spearman's rho correlation analysis revealed a statistically significant positive correlation between PSA levels and IPSS scores (r = 0.23, p = 0.00). This finding indicates that higher PSA levels tend to be associated with increased IPSS scores (Figure 2). These results underscore the importance of considering both PSA levels and IPSS scores in assessing prostate health and urinary function among individuals.

The Kruskal-Wallis test was conducted to assess the relationship between the DRE status groups and PSA levels (Table 4). The analysis revealed a statistically significant difference in PSA levels across different DRE status groups (p < 0.00). Comparisons indicated that participants with abnormal DRE findings had significantly higher mean PSA levels (M = 3.37 ng/mL, SD = 3.81) compared to those with normal DRE results (M = 2.00 ng/mL, SD = 1.96). Furthermore, PSA levels in the abnormal DRE group exhibited a wider range (0.01-31.15 ng/mL) compared to the normal DRE group. These findings suggest that abnormal DRE findings may be indicative of elevated PSA levels in this population, highlighting the potential utility of DRE in identifying individuals at risk of prostate abnormalities.

**Table 4:** Distribution of serum PSA levels according to the DRE status. Note: The data are represented as mean ± SD. A p-value of less than or equal to 0.001 was considered statistically significant. PSA: prostate-specific antigen; DRE: digital rectal examination.

DRE status	N	PSA mean (mean ± SD)	Std. deviation	Std. error	95% CI lower bound	95% CI upper bound	Min	Max	p-value at 95% CI
Normal	512	2.00 ± 1.96	1.96	0.09	1.83	2.17	0.01	19.08	
Abnormal	212	3.37 ± 3.81	3.81	0.26	2.85	3.88	0.01	31.15	
Total	724	2.40 ± 2.71	2.71	0.10	2.20	2.60	0.01	31.15	0.00

## Discussion

The primary issue with PCa is that it is often diagnosed too late and is usually asymptomatic in its early stages [25]. PSA testing remains the gold standard in screening for PCa early on [26]. Similar to previous studies [1,26], the present study found a significant correlation between PSA levels and age such that as age increases, the PSA levels increase. Due to the influence of factors such as race and age on PSA levels [27], establishing an age-specific reference range was essential in our Lebanese population to more accurately predict PCa, minimize false positives, and prevent overtreatment.

According to our findings, the mean PSA levels and the 95% CI were as follows: 1.74 (0.92-2.55) ng/mL for patients in the 50-54 years age group, 2.08 (1.79-2.37) ng/mL in the 55-59 years age group, 2.28 (1.94-2.62) ng/mL in the 60-64 years age group, 2.87 (2.39-3.34) ng/mL in the 65-69 years age group, and 2.65 (2.03-3.27) ng/mL in the above 70 years age group. The progressive increase in the mean PSA with the concurrent increase in age groups, in addition to the statistically significant positive correlation between age and PSA levels (r = 0.138, p < 0.01) all indicate that as age increases, there tends to be an increase in PSA levels among the participants. Previous findings further support this correlation [1,26]. However, the apparent drop in PSA mean from the 65-69 years age group to the above 70 years age group (Figure 1) could be attributed to low statistical power, due to the much lower number of patients in the above 70 years category (n = 63) compared to the 65-69 years group (n = 199). Future studies could include an equal number of participants in both age groups to explore the comparison further. Compared to our numbers, Reza et al.'s [1] mean PSA levels were as follows: “1.47 (1.26-1.68) ng/mL in the age group of 60-64 years, 1.63 (1.28-1.99) ng/mL in the age group of 65-69 years, 2.01 (1.47-2.54) ng/mL in the age group of 70-74 years.” Consequently, the difference in mean PSA levels compared to the Lebanese population indicates that race, in addition to age, has a significant effect on the level of PSA in patients.

As for LUTS and age, our analysis showed a significant relation between IPSS status groups and PSA levels such that participants classified as severely symptomatic exhibited higher mean PSA levels (M = 4.06 ng/mL, SD = 3.63) compared to those categorized as mildly symptomatic (M = 2.17 ng/mL, SD = 2.62) and moderately symptomatic (M = 2.07 ng/mL, SD = 2.16). Furthermore, we found a significant positive correlation between IPSS status groups and PSA levels (r = 0.23, p = 0.00). These findings indicate that higher PSA levels tend to be associated with increased IPSS scores (Figure 2). These results underscore the importance of considering both PSA levels and IPSS scores in assessing prostate health and urinary function among individuals. Our findings are also consistent with previous findings in regard to the relationship between IPSS and PSA [22,23]. Additionally, Patel et al. [28] concluded in their study that even men with mild/absent LUTS but with increased PSA are at a higher risk of developing incident LUTS possibly due to BPH. However, due to the various conditions that could cause LUTS (i.e., infections, BPH, and PCa), and the lack of data, the etiologies of these urinary symptoms are unknown. Future studies could build upon our work by categorizing the patients into different etiologies, which would require further screening and imaging tests.

Finally, we explored the relation between DRE status and PSA. DRE status groups were categorized into either normal or abnormal. Notably, age-related prostate growth was considered normal while nodular protrusions or excessive enlargement was considered abnormal. Abnormal DRE findings had significantly higher mean PSA levels (M = 3.37 ng/mL, SD = 3.81) compared to those with normal DRE results (M = 2.00 ng/mL, SD = 1.96). These findings suggest that abnormal DRE findings may be indicative of elevated PSA levels in this population, highlighting the potential utility of DRE in identifying individuals at risk of prostate abnormalities. These findings are in line with the NICE guidelines that push for DRE screening, and conflict with the AUA guidelines that doubt its usefulness altogether [6,8]. Additionally, our results are consistent with previous assessments of the relationship between DRE status and PSA, which found PSA levels to be elevated in positive DRE tests [5,10,11]. The consensus behind the opposition of some guidelines against DRE stems from its low clinical value in detecting PCa when PSA levels are normal [29]. Gosselaar et al. [30] found that abnormal DRE and PSA ≥ 3 ng/ml had an increased risk of PCa compared to those with normal DRE (positive predictive value 49% vs. 22%). Risk evaluation for PCa could be incorporated into upcoming studies that aim to replicate our findings to depict the sensitivity of DRE screening more accurately. This might include more decisive and invasive tests such as a transrectal biopsy or an MRI scan.

The findings of this study reveal important correlations between PSA levels, age, LUTS severity, and DRE results within the Lebanese population. These results are particularly significant given the unique context of Lebanon, where genetic predispositions, environmental factors such as diet and smoking prevalence, and distinct lifestyle factors may influence PSA levels differently than in Western populations. Additionally, the challenges posed by the Lebanese healthcare system, including disparities in access to preventive care and varying levels of health literacy, underscore the importance of our findings. Establishing age-specific PSA reference ranges tailored to Lebanese men could enhance the accuracy of PCa screening, thereby reducing the risks of overdiagnosis and underdiagnosis. Furthermore, our results support the need for comprehensive public health campaigns to raise awareness about prostate health and improve access to screening, particularly in underserved areas. Integrating PSA testing with clinical assessments, such as DRE and LUTS evaluation, could further optimize early detection and improve patient outcomes in this population.

Other noteworthy limitations include the selection bias mentioned previously, which stems from the fact that our participants were volunteers. Additionally, this study's findings may have limited generalizability beyond the Lebanese population and the specific age range studied.

## Conclusions

In conclusion, our study highlights the critical importance of developing age-specific reference ranges for PSA levels tailored to the Lebanese population, which are essential for accurate PCa prediction and reducing the risk of false positives. The significant positive association between age and PSA levels emphasizes the need for these tailored reference ranges. Additionally, the observed correlations between PSA levels, LUTS, and abnormal DRE findings underscore the necessity for a comprehensive assessment in clinical practice. Given the unique genetic, environmental, and lifestyle factors prevalent in Lebanon, along with disparities in healthcare access and varying levels of health literacy, it is crucial to integrate multiple clinical parameters, including PSA levels, age, LUTS, and DRE findings, to enhance prostate health assessment and optimize patient care in this population. This approach will not only improve early detection but also address the specific healthcare challenges faced by Lebanese men, ultimately leading to better PCa outcomes.
